# Supplementary material for: HIVprotI: an integrated web based platform for prediction and design of HIV proteins inhibitors
Source: J Cheminform. 2018 Mar 9;10:12. doi: 10.1186/s13321-018-0266-y (PMC5845081; doi:10.1186/s13321-018-0266-y)
Supplement: Supplementary file 2 — Additional file 2. Source code of HIVProtI web server. [file 13321_2018_266_MOESM2_ESM.zip › HIVprotI_Source-code/hivprotI/weka-3-6-6/doc/weka/core/TechnicalInformation.Field.html]

TechnicalInformation.Field


---


|  |  |  |
| --- | --- | --- |
| **Overview** \| **Package** \| **Class** \| **Tree** \| **Deprecated** \| **Index** \| **Help** \| **Weka's home** | |  |
| **PREV CLASS**   **NEXT CLASS** | **FRAMES**    **NO FRAMES**     **All Classes** |  |
| SUMMARY: NESTED \| ENUM CONSTANTS \| FIELD \| METHOD | DETAIL: ENUM CONSTANTS \| FIELD \| METHOD |  |


---


## weka.core Enum TechnicalInformation.Field

```
java.lang.Object
  java.lang.Enum<TechnicalInformation.Field>
      weka.core.TechnicalInformation.Field
```

**All Implemented Interfaces:**: java.io.Serializable, java.lang.Comparable<TechnicalInformation.Field>

**Enclosing class:**: TechnicalInformation

---

``` public static enum TechnicalInformation.Field extends java.lang.Enum<TechnicalInformation.Field> ```

the possible fields

---

**Enum Constant Summary**

`ABSTRACT`
  
          An abstract of the work.

`ADDRESS`
  
          Usually the address of the publisher or other type of institution.

`AFFILIATION`
  
          The authors affiliation.

`ANNOTE`
  
          An annotation.

`AUTHOR`
  
          The name(s) of the author(s), in the format described in the LaTeX book.

`BOOKTITLE`
  
          Title of a book, part of which is being cited.

`CHAPTER`
  
          A chapter (or section or whatever) number.

`CONTENTS`
  
          A Table of Contents.

`COPYRIGHT`
  
          Copyright information.

`CROSSREF`
  
          The database key of the entry being cross referenced.

`EDITION`
  
          The edition of a book---for example, ``Second''.

`EDITOR`
  
          Name(s) of editor(s), typed as indicated in the LaTeX book.

`HOWPUBLISHED`
  
          How something strange has been published.

`HTTP`
  
          A link to a postscript file.

`INSTITUTION`
  
          The sponsoring institution of a technical report.

`ISBN`
  
          The International Standard Book Number (10 digits).

`ISBN13`
  
          The International Standard Book Number (13 digits).

`ISSN`
  
          The International Standard Serial Number.

`JOURNAL`
  
          A journal name.

`KEY`
  
          Used for alphabetizing, cross referencing, and creating a label when the ``author'' information is missing.

`KEYWORDS`
  
          Key words used for searching or possibly for annotation.

`LANGUAGE`
  
          The language the document is in.

`LCCN`
  
          The Library of Congress Call Number.

`LOCATION`
  
          A location associated with the entry, such as the city in which a conference took place.

`MONTH`
  
          The month in which the work was published or, for an unpublished work, in which it was written.

`MRNUMBER`
  
          The Mathematical Reviews number.

`NOTE`
  
          Any additional information that can help the reader.

`NUMBER`
  
          The number of a journal, magazine, technical report, or of a work in a series.

`ORGANIZATION`
  
          The organization that sponsors a conference or that publishes a manual.

`PAGES`
  
          One or more page numbers or range of numbers, such as 42--111 or 7,41,73--97 or 43+ (the `+' in this last example indicates pages following that don't form a simple range).

`PDF`
  
          A link to a postscript file.

`PRICE`
  
          The price of the document.

`PS`
  
          A link to a postscript file.

`PUBLISHER`
  
          The publisher's name.

`SCHOOL`
  
          The name of the school where a thesis was written.

`SERIES`
  
          The name of a series or set of books.

`SIZE`
  
          The physical dimensions of a work.

`TITLE`
  
          The work's title, typed as explained in the LaTeX book.

`TYPE`
  
          The type of a technical report---for example, ``Research Note''.

`URL`
  
          The WWW Universal Resource Locator that points to the item being referenced.

`VOLUME`
  
          The volume of a journal or multi-volume book.

`YEAR`
  
          The year of publication or, for an unpublished work, the year it was written.


| **Method Summary** | |
| --- | --- |
| `java.lang.String` | `getComment()`             returns the comment string |
| `java.lang.String` | `getDisplay()`             returns the display string |
| `java.lang.String` | `toString()`             returns the display string of the Type |
| `static TechnicalInformation.Field` | `valueOf(java.lang.String name)`             Returns the enum constant of this type with the specified name. |
| `static TechnicalInformation.Field[]` | `values()`             Returns an array containing the constants of this enum type, in the order they are declared. |

**Methods inherited from class java.lang.Enum**

`compareTo, equals, getDeclaringClass, hashCode, name, ordinal, valueOf`

**Methods inherited from class java.lang.Object**

`getClass, notify, notifyAll, wait, wait, wait`

**Enum Constant Detail**

### ADDRESS

```
public static final TechnicalInformation.Field ADDRESS
```

:   Usually the address of the publisher or other type of institution. For major publishing houses, van Leunen recommends omitting the information entirely. For small publishers, on the other hand, you can help the reader by giving the complete address.

---


### ANNOTE

```
public static final TechnicalInformation.Field ANNOTE
```

:   An annotation. It is not used by the standard bibliography styles, but may be used by others that produce an annotated bibliography.

---


### AUTHOR

```
public static final TechnicalInformation.Field AUTHOR
```

:   The name(s) of the author(s), in the format described in the LaTeX book.

---


### BOOKTITLE

```
public static final TechnicalInformation.Field BOOKTITLE
```

:   Title of a book, part of which is being cited. See the LaTeX book for how to type titles. For book entries, use the title field instead.

---


### CHAPTER

```
public static final TechnicalInformation.Field CHAPTER
```

:   A chapter (or section or whatever) number.

---


### CROSSREF

```
public static final TechnicalInformation.Field CROSSREF
```

:   The database key of the entry being cross referenced. Any fields that are missing from the current record are inherited from the field being cross referenced.

---


### EDITION

```
public static final TechnicalInformation.Field EDITION
```

:   The edition of a book---for example, ``Second''. This should be an ordinal, and should have the first letter capitalized, as shown here; the standard styles convert to lower case when necessary.

---


### EDITOR

```
public static final TechnicalInformation.Field EDITOR
```

:   Name(s) of editor(s), typed as indicated in the LaTeX book. If there is also an author field, then the editor field gives the editor of the book or collection in which the reference appears.

---


### HOWPUBLISHED

```
public static final TechnicalInformation.Field HOWPUBLISHED
```

:   How something strange has been published. The first word should be capitalized.

---


### INSTITUTION

```
public static final TechnicalInformation.Field INSTITUTION
```

:   The sponsoring institution of a technical report.

---


### JOURNAL

```
public static final TechnicalInformation.Field JOURNAL
```

:   A journal name. Abbreviations are provided for many journals.

---


### KEY

```
public static final TechnicalInformation.Field KEY
```

:   Used for alphabetizing, cross referencing, and creating a label when the ``author'' information is missing. This field should not be confused with the key that appears in the cite command and at the beginning of the database entry.

---


### MONTH

```
public static final TechnicalInformation.Field MONTH
```

:   The month in which the work was published or, for an unpublished work, in which it was written. You should use the standard three-letter abbreviation, as described in Appendix B.1.3 of the LaTeX book.

---


### NOTE

```
public static final TechnicalInformation.Field NOTE
```

:   Any additional information that can help the reader. The first word should be capitalized.

---


### NUMBER

```
public static final TechnicalInformation.Field NUMBER
```

:   The number of a journal, magazine, technical report, or of a work in a series. An issue of a journal or magazine is usually identified by its volume and number; the organization that issues a technical report usually gives it a number; and sometimes books are given numbers in a named series.

---


### ORGANIZATION

```
public static final TechnicalInformation.Field ORGANIZATION
```

:   The organization that sponsors a conference or that publishes a manual.

---


### PAGES

```
public static final TechnicalInformation.Field PAGES
```

:   One or more page numbers or range of numbers, such as 42--111 or 7,41,73--97 or 43+ (the `+' in this last example indicates pages following that don't form a simple range). To make it easier to maintain Scribe-compatible databases, the standard styles convert a single dash (as in 7-33) to the double dash used in TeX to denote number ranges (as in 7--33).

---


### PUBLISHER

```
public static final TechnicalInformation.Field PUBLISHER
```

:   The publisher's name.

---


### SCHOOL

```
public static final TechnicalInformation.Field SCHOOL
```

:   The name of the school where a thesis was written.

---


### SERIES

```
public static final TechnicalInformation.Field SERIES
```

:   The name of a series or set of books. When citing an entire book, the the title field gives its title and an optional series field gives the name of a series or multi-volume set in which the book is published.

---


### TITLE

```
public static final TechnicalInformation.Field TITLE
```

:   The work's title, typed as explained in the LaTeX book.

---


### TYPE

```
public static final TechnicalInformation.Field TYPE
```

:   The type of a technical report---for example, ``Research Note''.

---


### VOLUME

```
public static final TechnicalInformation.Field VOLUME
```

:   The volume of a journal or multi-volume book.

---


### YEAR

```
public static final TechnicalInformation.Field YEAR
```

:   The year of publication or, for an unpublished work, the year it was written. Generally it should consist of four numerals, such as 1984, although the standard styles can handle any year whose last four nonpunctuation characters are numerals, such as `\\hbox{(about 1984)}'.

---


### AFFILIATION

```
public static final TechnicalInformation.Field AFFILIATION
```

:   The authors affiliation.

---


### ABSTRACT

```
public static final TechnicalInformation.Field ABSTRACT
```

:   An abstract of the work.

---


### CONTENTS

```
public static final TechnicalInformation.Field CONTENTS
```

:   A Table of Contents.

---


### COPYRIGHT

```
public static final TechnicalInformation.Field COPYRIGHT
```

:   Copyright information.

---


### ISBN

```
public static final TechnicalInformation.Field ISBN
```

:   The International Standard Book Number (10 digits).

---


### ISBN13

```
public static final TechnicalInformation.Field ISBN13
```

:   The International Standard Book Number (13 digits).

---


### ISSN

```
public static final TechnicalInformation.Field ISSN
```

:   The International Standard Serial Number. Used to identify a journal.

---


### KEYWORDS

```
public static final TechnicalInformation.Field KEYWORDS
```

:   Key words used for searching or possibly for annotation.

---


### LANGUAGE

```
public static final TechnicalInformation.Field LANGUAGE
```

:   The language the document is in.

---


### LOCATION

```
public static final TechnicalInformation.Field LOCATION
```

:   A location associated with the entry, such as the city in which a conference took place.

---


### LCCN

```
public static final TechnicalInformation.Field LCCN
```

:   The Library of Congress Call Number. I've also seen this as lib-congress.

---


### MRNUMBER

```
public static final TechnicalInformation.Field MRNUMBER
```

:   The Mathematical Reviews number.

---


### PRICE

```
public static final TechnicalInformation.Field PRICE
```

:   The price of the document.

---


### SIZE

```
public static final TechnicalInformation.Field SIZE
```

:   The physical dimensions of a work.

---


### URL

```
public static final TechnicalInformation.Field URL
```

:   The WWW Universal Resource Locator that points to the item being referenced. This often is used for technical reports to point to the ftp site where the postscript source of the report is located.

---


### PS

```
public static final TechnicalInformation.Field PS
```

:   A link to a postscript file.

---


### PDF

```
public static final TechnicalInformation.Field PDF
```

:   A link to a postscript file.

---


### HTTP

```
public static final TechnicalInformation.Field HTTP
```

:   A link to a postscript file.


**Method Detail**

### values

```
public static TechnicalInformation.Field[] values()
```

:   Returns an array containing the constants of this enum type, in
    the order they are declared. This method may be used to iterate
    over the constants as follows:

    ```
    for (TechnicalInformation.Field c : TechnicalInformation.Field.values())
        System.out.println(c);
    ```

    :   **Returns:**: an array containing the constants of this enum type, in the order they are declared

---


### valueOf

```
public static TechnicalInformation.Field valueOf(java.lang.String name)
```

:   Returns the enum constant of this type with the specified name.
    The string must match *exactly* an identifier used to declare an
    enum constant in this type. (Extraneous whitespace characters are
    not permitted.)

    :   **Parameters:**: `name` - the name of the enum constant to be returned. **Returns:**: the enum constant with the specified name **Throws:**: `java.lang.IllegalArgumentException` - if this enum type has no constant with the specified name: `java.lang.NullPointerException` - if the argument is null

---


### getDisplay

```
public java.lang.String getDisplay()
```

:   returns the display string

    :   **Returns:**: the display string

---


### getComment

```
public java.lang.String getComment()
```

:   returns the comment string

    :   **Returns:**: the comment string

---


### toString

```
public java.lang.String toString()
```

:   returns the display string of the Type

    :   **Overrides:**: `toString` in class `java.lang.Enum<TechnicalInformation.Field>`
    :   **Returns:**: the display string


---


|  |  |  |
| --- | --- | --- |
| **Overview** \| **Package** \| **Class** \| **Tree** \| **Deprecated** \| **Index** \| **Help** \| **Weka's home** | |  |
| **PREV CLASS**   **NEXT CLASS** | **FRAMES**    **NO FRAMES**     **All Classes** |  |
| SUMMARY: NESTED \| ENUM CONSTANTS \| FIELD \| METHOD | DETAIL: ENUM CONSTANTS \| FIELD \| METHOD |  |


---
